# Optogenetically Blocking Sharp Wave Ripple Events in Sleep Does Not Interfere with the Formation of Stable Spatial Representation in the CA1 Area of the Hippocampus

**DOI:** 10.1371/journal.pone.0164675

**Published:** 2016-10-19

**Authors:** Krisztián A. Kovács, Joseph O’Neill, Philipp Schoenenberger, Markku Penttonen, Damaris K. Ranguel Guerrero, Jozsef Csicsvari

**Affiliations:** 1 Institute of Science and Technology Austria, Klosterneuburg, Austria; 2 Department of Psychology, University of Jyvaskyla, Jyvaskyla, Finland; University of Oxford, UNITED KINGDOM

## Abstract

During hippocampal sharp wave/ripple (SWR) events, previously occurring, sensory input-driven neuronal firing patterns are replayed. Such replay is thought to be important for plasticity-related processes and consolidation of memory traces. It has previously been shown that the electrical stimulation-induced disruption of SWR events interferes with learning in rodents in different experimental paradigms. On the other hand, the cognitive map theory posits that the plastic changes of the firing of hippocampal place cells constitute the electrophysiological counterpart of the spatial learning, observable at the behavioral level. Therefore, we tested whether intact SWR events occurring during the sleep/rest session after the first exploration of a novel environment are needed for the stabilization of the CA1 code, which process requires plasticity. We found that the newly-formed representation in the CA1 has the same level of stability with optogenetic SWR blockade as with a control manipulation that delivered the same amount of light into the brain. Therefore our results suggest that at least in the case of passive exploratory behavior, SWR-related plasticity is dispensable for the stability of CA1 ensembles.

## Introduction

Hippocampal SWR events predominantly occur during slow-wave sleep and immobile epochs in waking[1]. They are recognized by two particular local field potential patterns: sharp waves showing a strong negative deflection in the *stratum radiatum* of the CA1 region caused by the population EPSP events triggered in the dendrites of the pyramidal cells, and high frequency (can reach200Hz) ripple oscillations[2] that are conspicuous in the *stratum pyramidale* of the CA1 region[3]. It has been demonstrated that hippocampal SWR events are highly synchronized both within and between the hemispheres. They are thought to convey information to downstream cortical target areas while other subcortical structures are silent[4]. They provide also a framework for the replay of behaviorally-relevant information encountered in preceding waking periods, such as the replay of sequences of recently visited places by CA1 place cells. In addition to simply subserving consolidation-related processes, a recent report points to the existence of cognitive-like processing of spatial information and provides evidence that trajectories containing never explored shortcuts are also expressed in SWR envelopes during slow-wave sleep[5]. This raises the possibility that SWR events contribute to higher order cognitive processes.

In line with their proposed role in memory consolidation and cognitive processes, SWR events have been shown to be altered in rodent models of neuropsychiatric disorders. Various alterations of SWR events were reported in different transgenic mouse models of dementia. Specifically, in an apolipoprotein-E4-based genetic model, their abundance was decreased[6] while, in a tauopathy-based genetic model, lower amplitude ripple oscillation and altered temporal structure was reported[7]. Interestingly, when applied exogenously, β-amyloid oligomers affected the post-encoding peaks of SWR occurrence only in mice that had performed a cognitively challenging task, while leaving the baseline occurrence unaltered[8].

Disruption of the SWR events by electrical stimulation of the ventral hippocampal commissure has provided the first line of evidence that the consolidation of task-related memories is linked to the SWR periods of slow-wave sleep. Specifically, electric SWR-blockade in post-training slow-wave sleep interfered with learning and decreased the performance in an 8-arm radial maze task[9]. In a similar experiment, SWR-blocking stimulation was applied only in the post-sleeps following one specific maze configuration and not for the mirrored version of the same maze. When the performance in the 2 maze configurations were compared, the results showed that the animals could find their way less in the maze for which ripple blockade was applied[10]. SWR events also occur during brief pauses in exploration. These events are in all aspects similar to those occurring in slow-wave sleep and have been termed awake or exploratory SWR events (eSWR events)[11]. Blocking the eSWR events while the rodents learn a W-maze task interferes with the gradual improvement of the behavioral performance related to the working memory component but had not effect on the reference memory [12].

Given the convincing evidence regarding the need of SWR events in sleep for memory stabilization and subsequent successful recall, we have embarked on studying the effect of SWR-blockade on the ensemble spatial code in hippocampal area CA1. Such code has been observed to change in concordance with behavioral performance[13] and is generally supposed to underlie behavioral improvement in all tasks where there is a spatial demand.

All previous studies aiming at disrupting SWR events used electrical stimulation that lacks regional and cell type specificity. Therefore, in the present work we decided to take advantage of the recent improvements of the optogenetic tools, and we used laser light pulses instead of the electric stimulation to disrupt SWR events. Correspondingly, we have carried out all of the experiments in mice expressing archaerhodopsin (Arch-EGFP) in the pyramidal cells of the CA1 area and lacking expression in CA3 and in other cell types of the CA1. These animals have been obtained by crossing a CaMKII-Cre driver mouse [14] line with a mouse line harboring a Cre-dependent Arch-EGFP gene [15] (see methods).

## Results

To assess the change in the spatial code between two subsequent exposures to the same, initially novel environment, we have implanted three archaerhodopsin expressing C57/BL6 mice with microdrives harboring 15 tetrodes (7 independently movable tetrode pairs and one independently movable tetrode, S1 Fig) to record and to silence the dorsal CA1 region. The expression of the Arch-EGFP construct was confirmed in hippocampal sections from littermates (S2 Fig). These sections showed high level of the exogenous protein in the cell membranes of the granule cells in the dentate gyrus and in those of the principal cells in CA1, but with no expression in the CA3 area, and low expression levels in the cortex. In order to block SWR events bilaterally in the CA1 area, 4 conical optic fibers (2 per hemisphere) were incorporated in the microdrives in a fixed position (S1 and S3 Figs) The tips of the fibers were targeted into the *stratum oriens*, 150 μm above the *stratum pyramidale* of the CA1. The layout of tetrodes and optic fibers covered the full dorsal CA1 region of the mouse (S4 Fig).

**Fig 1:**
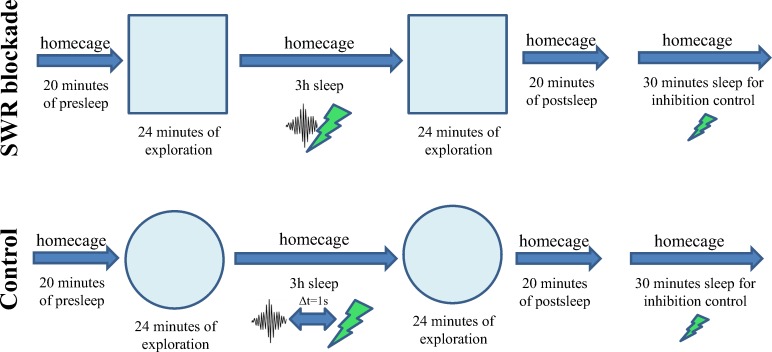
Details of the experimental paradigm. New environments (different shape, and different texture of side-walls) were used on each day, the square box and the circular arena are only shown as examples. Laser pulses were delayed by 1.32 s on control days. Regular, half-intensity laser pulses were used during the final sleep/rest to verify the efficiency of the optogenetic inhibition.

**Fig 2:**
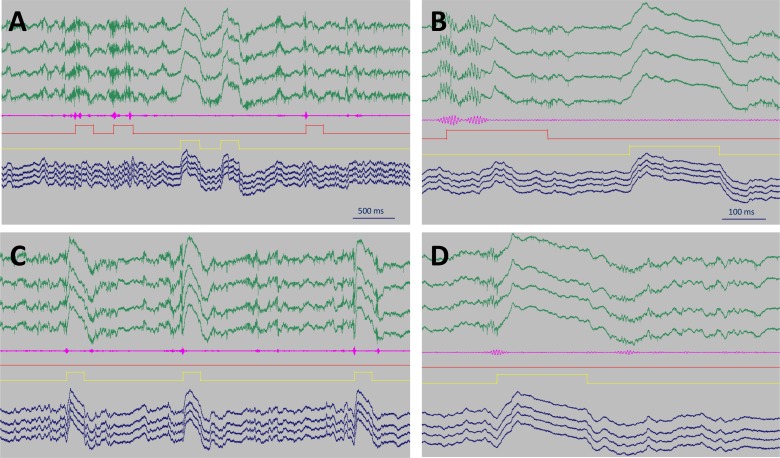
**SWR detection and disruption (A, B)** In the control condition, ripple oscillations (filtered differential signal in magenta) triggered a 200 ms long detector signal (red square pulses) that was fed back into the recording computer and delayed by 1.32 s. The delayed signals (yellow square pulses) drove the laser and elicited field responses. **(C, D)** In the SWR-blockade condition, the signal coming from the detector directly drove the laser (yellow square pulses) and readily destroyed the ripples. On all of the panels **(A, B, C, D)** a tetrode positioned in the CA1 layer with unit activity is shown (green), and another one positioned below (blue). Field responses are obvious on all traces. Lack of inhibition in the interneurons explains the residual unit activity during field responses.

At the beginning of each recording day, a pre-sleep session was recorded in the home cage followed by 24 minutes of exploration in a novel enclosure that the animal have not experienced before. Subsequently animals were allowed to sleep in their cage for 3 hours before the second 24-minutes-long exploration of the same enclosure, and finally a post-sleep session was recorded in the home cage (Fig 1). In half of the experiments, referred to as ***SWR-blockade condition***, 200 ms long green laser pulses were triggered by the detection of the onset of the ripple event during the 3 hour long middle sleep/rest sessions. The other half of the recording days were assigned to the ***control condition;*** on these days a 1.32 s delay was introduced between the detected ripple event and the trigger pulse that drove the laser. In this way the same amount of light-induced suppression (and therefore, the same amount of energy) was delivered to the brain on the control days as on the days when SWR events in sleep/rest were blocked (Fig 2).

In order to detect the onset of SWR events, the currently recorded band-pass filtered differential signal (generated by subtracting a channel in the *stratum radiatum* from a channel in the *stratum pyramidale* to isolate ripple oscillations) was fed into an analogue ripple detector device that sent a 200 ms long trigger signal to a laser source whenever the amplitude of the band-passed filtered signal exceeded a threshold. Upon triggering, 561 nm laser light was delivered into the CA1 region via an optic cable branching into 4 daughter cables via a rotatory joint to irradiate the 4 contact points on the microdrive.

Examples of trigger signals and light pulses are shown in Fig 2. Strong, in a few instances saturating field responses were observed during light pulses on all tetrodes, and the oscillation of the field was destroyed after at maximum two ripple cycles in the SWR-blockade condition (Fig 2C and 2D) In the control condition, SWRs triggered a 200 ms long detector signal that, in turn, set off a delayed signal driving the laser. Thus the detector signal frequently covered more than one SWR event (Fig 2A and 2B).

**Fig 3:**
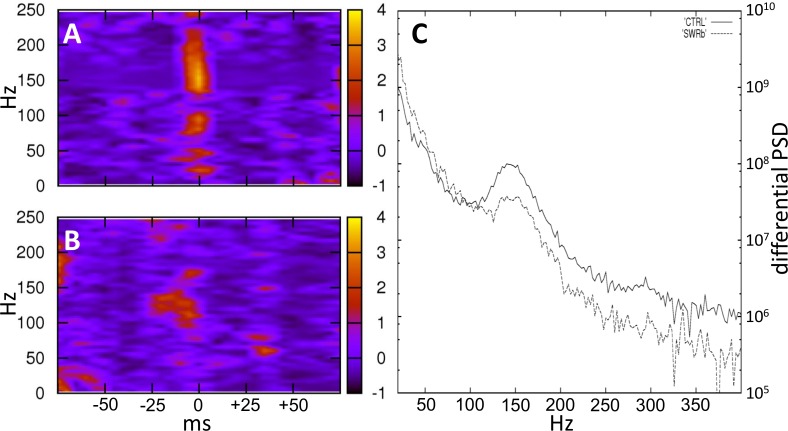
**Vanishing high frequency oscillatory activity in the SWR-blockade condition (A)** In the control condition, light-time triggered power spectrum shows a prominent peak at 150 Hz and higher frequencies. Here all the data points were shifted forward by 1.32 s, so that the SWR event that actually triggered the light appear at time 0. **(B)** In the SWR-blockade condition, virtually no oscillatory activity is observable above 150 Hz around the onset of the laser pulse. **(C)** Normalized power spectrum around off-line detected SWR events. To perform the normalization, spectra were calculated using the multi-taper method around the events, then around time points shifted by 400 ms, and the latter was subtracted from the former. In total, 643 off-line detected SWR events were used for the control condition (continuous line) and 348 for the SWR-blockade condition (dashed line). All of the data presented here **(A, B, C)** were from the first 20 minutes of the middle sleep and from one example tetrode for each of the conditions. The other tetrodes showed similar activity.

We generated power spectra to check how efficiently SWR events are eliminated (Fig 3). Using 150 ms long time windows of power spectra around the time points when the on-line detector sensed an SWR event, we could visualize that in the control condition oscillatory activity around 160 Hz was predominant (Fig 3A), while in the SWR-blockade condition, oscillation was abolished (Fig 3B). To take into consideration that some of the SWR events might have been undetected–although no evidence of this was seen in the raw traces–we have also used a non-stringent offline SWR detector. Low stringency made sure that the truncated, low amplitude portions of SWR events could be identified as oscillatory events in the SWR-blockade conditions, and time points of the events could also be provided offline independently of our on-line detector. When calculating normalized power spectra around the off-line detected time points using the multi-taper method, the decrease of the oscillatory activity around 150 Hz and higher frequencies was evident (Fig 3C).

**Fig 4:**
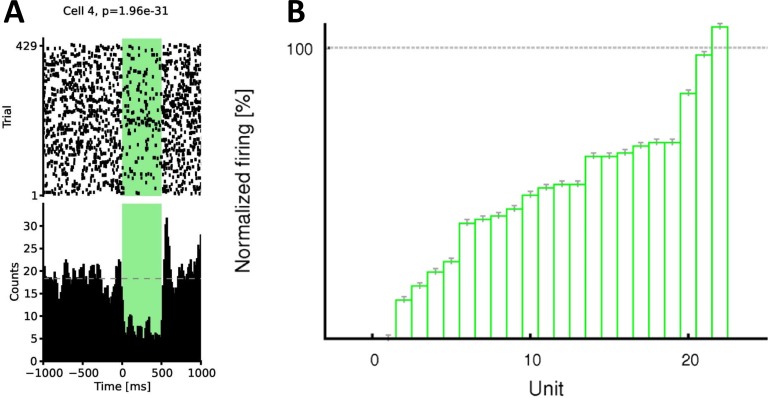
Efficiency of the inhibition. **(A)** Efficiency of the optogenetic inhibition of one single CA1 pyramidal cell. Regularly timed 500 ms long half-intensity light pulses were delivered during the final 30 minute long sleep/rest of the day. Spiking activity is displayed as a raster plot centered around the onset of the light pulse (left) and as histogram using 10 ms bins (right). Rebound activity after inhibition can also be observed on the histogram. P-value derived from the Wilcoxon signed rank sum test is shown above the raster. **(B)** Efficiency of the optogenetic inhibition at population level (22 pyramidal cells are shown from one example recording day). Similarly to other recording days, about 90% of all pyramidal cells were significantly inhibited by the regular 500 ms light pulses.

To check the inhibition at single cell level, on each recording day, after the second post-sleep, a further sleep session was recorded with regularly timed laser pulses of half intensity to quantify the efficiency of the inhibition for each cell. Using the method presented in a previous study from our lab [16] we have found that over 90% of the pyramidal cells were significantly inhibited, and none of them were disinhibited. An example set of 22 cells from one recording day is shown in Fig 2A. Reducing the intensity to half guaranteed that the light-evoked field responses did not cause saturation and hence the unit activity could reliably be quantified also during the laser pulses. The effect of optogenetic inhibition of a single recording day at the population level is shown in Fig 4B. The efficiency of the inhibition should have been at least the same but most probably higher when the SWR events were targeted, given that then the intensity of the laser was twice as high as in Fig 4.

**Fig 5:**
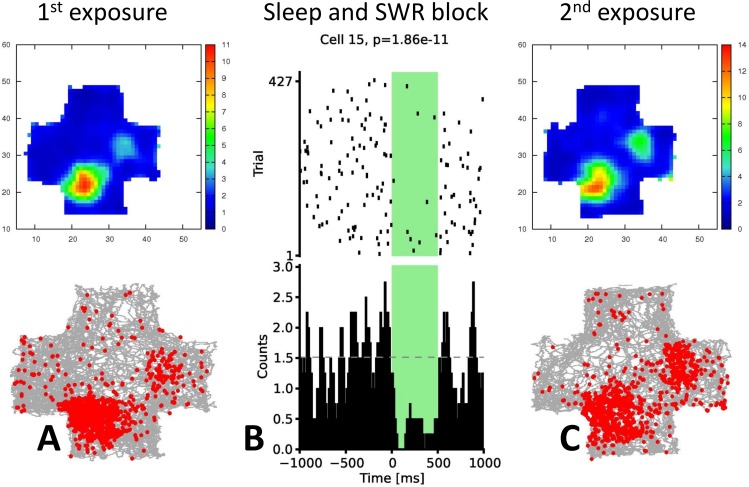
Stability of one single inhibited CA1 pyramidal cell on a day with SWR blockade. **(A)** the place field within the enclosure and the maximum firing rate and the spikes plotted on the trajectory of the animal are shown for the cell before the optogenetic intervention **(C)** and thereafter. **(B)** The raster plot and the histogram shows the efficiency of the inhibition. Neither the location of the strongest spiking activity nor the location of the place field in the cross-maze change substantially.

**Fig 6:**
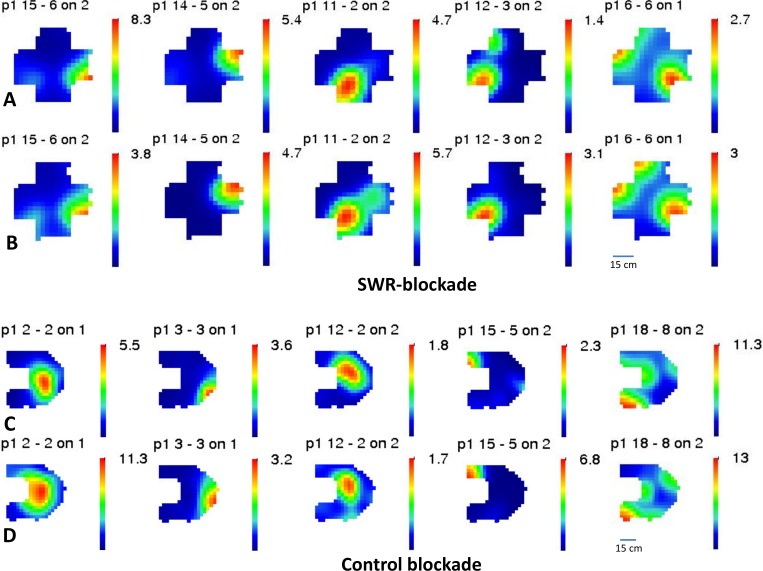
Stability of the firing rate maps. A selected set of 10 CA1 pyramidal cells, recorded on an SWR-blockade day **(A, B)** and on a control day **(C, D)** is shown. For each day, the upper row shows the rate maps before inhibition **(A, C),** the lower one the rate maps after inhibition **(B, D).** Note the substantial level of stability on SWR-blockade days.

The stability of spatial representation was assessed by constructing firing rate maps for the first and second exposure to the same environment for each of the recorded CA1 pyramidal cells. A representative cell on a day with SWR blockade is shown in Fig 5. In spite of SWR-locked optogenetic inhibition in the middle sleep/rest (Fig 5B), the cell had stable firing properties and a stable place field before (Fig 5A) and after (Fig 5C) the SWR-blockade. At the population level, some changed the location of their place field while some had their highest activity at the same location, with a high number of stable cells on days with SWR blockade (Fig 6).

**Fig 7:**
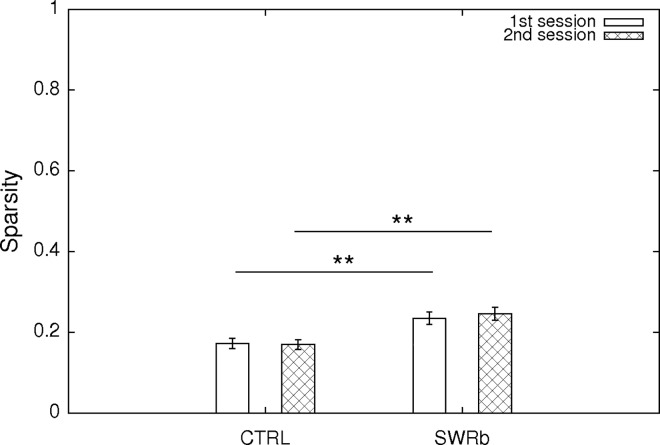
Sparsity of the cells. Mean ± SE of the sparsity of the CA1 pyramidal neurons at first exposure (open bars) and second exposure (cross-hatched bars) to the same environment. Sparsities at the first exploration session are significantly different between the control and SWR-block condition (p = 0.004; Oneway ANOVA), as well as for the second exploration session (p = 0.002; Oneway ANOVA). 117 (SWR block) + 116 (CTRL) pyramidal cells were used for this analysis.

**Fig 8:**
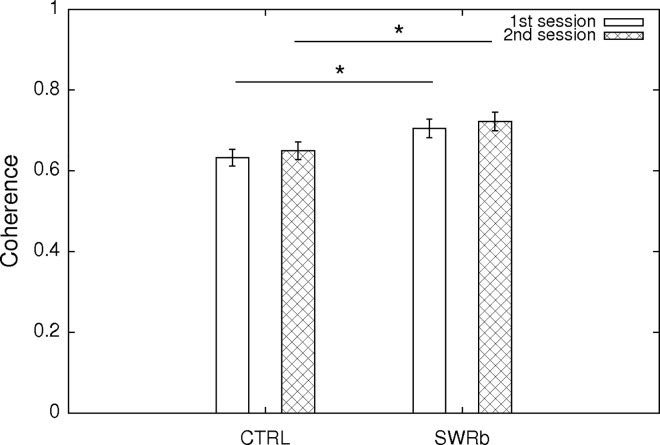
Coherence of the cells. Mean ± SE of the coherence of the CA1 pyramidal neurons at first exposure (open bars) and second exposure (cross-hatched bars) to the same environment. Coherence values at the first exploration session are significantly different between the control and SWR-block condition (p = 0.005; Oneway ANOVA), and the similarly for the second exploration session (p = 0.012; Oneway ANOVA) 117 (SWR block) + 116 (CTRL) pyramidal cells were used for this analysis.

For each principal neuron, in order to quantify the quality of their place-related firing, we have first calculated the sparsity and coherence of the firing rate maps, which describe, respectively, how restrictedly in space they fire and how continuous their place field is. Although both sparsity and coherence values were significantly different (all p < 0.05) between the control group and SWR-blockade group (as shown in Fig 7 and Fig 8), such effect was already present before the optogenetic intervention at the first exposure, and the magnitude of the effect did not change after the SWR-disruption applied in sleep/rest. Therefore, we attribute such difference to the fact that the two groups of animals have experienced different sets of environments due to the necessity of presenting new (unvisited) environments on each recording day. Slightly different spatial properties of the cells could be accounted for by slightly different edge lengths and dissimilar geometry of the different environments. Nevertheless we still note that the mean of the recorded sparsity and coherence values were in all cases within the range of what is being considered as normal (unaltered) for CA1 pyramidal cells[17].

**Fig 9:**
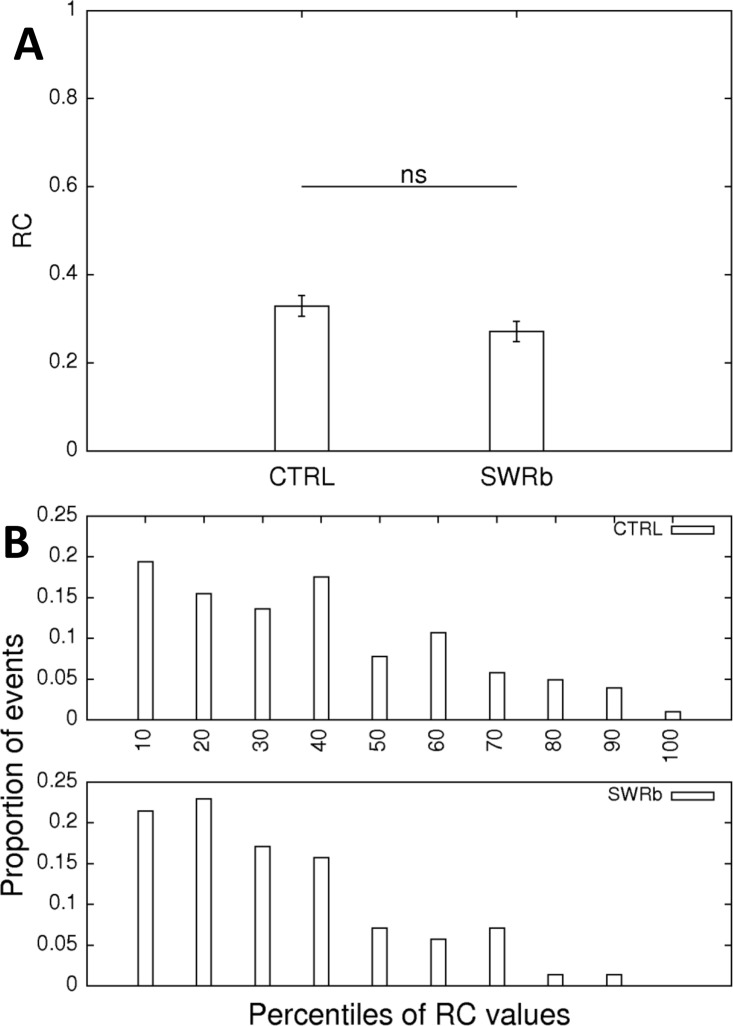
Changes of the average firing rates of the cells. **(A)** Firing rate changes (RC) between the first and second exploration of the same environment according to the formula defined in the text, on control days (left bar) and SWR-blockade days (right bar). The difference between the two RC values is not significant (p = 0.092, Oneway ANOVA). **(B)** The distribution of such changes is shown as a histogram in decimal steps along all possible rate change values. The two distributions are not significantly different (Kolmogorov-Smirnov test; p = 0.179; D = 0.167). 117 (SWR block) + 116 (CTRL) pyramidal cells were used for this analysis.

One prominent feature of the CA1 code is the remapping of the place fields between two exploration sessions. While remapping is robust when two different environments are used, it can signal the instability of the code if the animal is exposed to the same enclosure twice. Remapping can be termed global when the strongest firing of a particular neuron is observable at a completely different position, a new place field emerges or a former one disappears. Remapping can also be restricted to a significant change of the peak firing rate of a neuron while the place field stays at the same location. Therefore we have first calculated the firing rate changes (RC) of the individual cells between the two explorations using the formula RC = |(*f*_1_-*f*_2_)|/(*f*_1_+*f*_2_) which is meant to cover both rate remapping and global remapping, since global remapping can also be accompanied by a rate changes. We could not show any significant change at the ensemble level (p = 0.092; Fig 9A), the population undergoing SWR-blockade even had slightly (non-significantly) more stable firing rates. The histograms of the rate-change values for the control and the SWR-blockade conditions are similar and show that the more stable cells are somewhat overrepresented in both conditions (Fig 9B).

**Fig 10:**
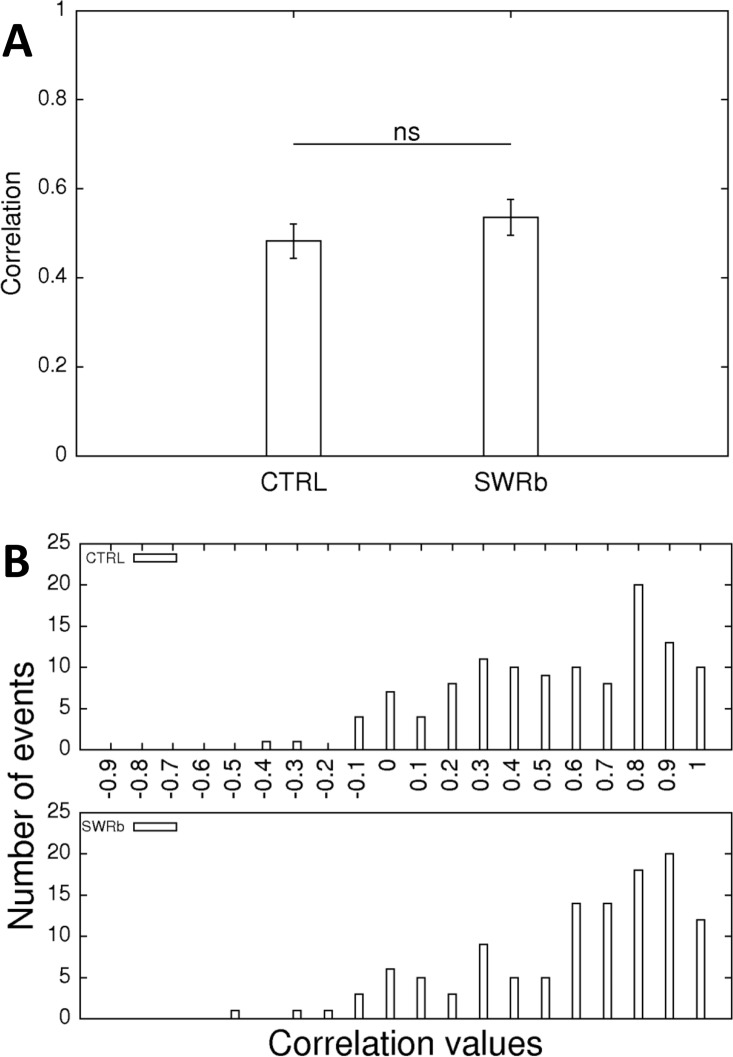
Stability of the firing rate maps. **(A)** Pearson correlation of the firing rate maps between the first and second exploration of the same environment on control days (left bar) and SWR-blockade days (right bar). No significant difference is detected (p = 0.334; Oneway ANOVA). **(B)** The distribution of such changes is shown as a histogram using bins of 0.1 along all possible correlation values. The two distributions are not significantly different (Kolmogorov-Smirnov test; p = 0.983; D = 0.10).

To further characterize the stability of the representation, we compared the firing rate maps for the first and second exploration sessions. For this we have calculated the Pearson correlation values between the rate values of the bins at first exposure and the rate values of the congruent bins at the second exposure to the same environment. An identical pair of firing rate maps would yield a correlation value of 1 while a completely new, unrelated spatial representation would yield a correlation value of 0. When considering all the CA1 pyramidal cells, a mean correlation value around 0.5 is obtained for both conditions, typical for the mouse and for 3 hours elapsing between the 2 exposures. The mean correlation values were not different for SWR-blockade and control experiments (Fig 10A). Furthermore, the distribution of the correlation values did not differ significantly either (Kolmogorov-Smirnov test; p = 0.983; D = 0.10, Fig 10B). For both conditions two subpopulations of cells could be suspected: that of more stable (correlations around 0.8–0.9) and less stable (correlations around 0.3–0.4) neurons (Fig 10B).

**Fig 11:**
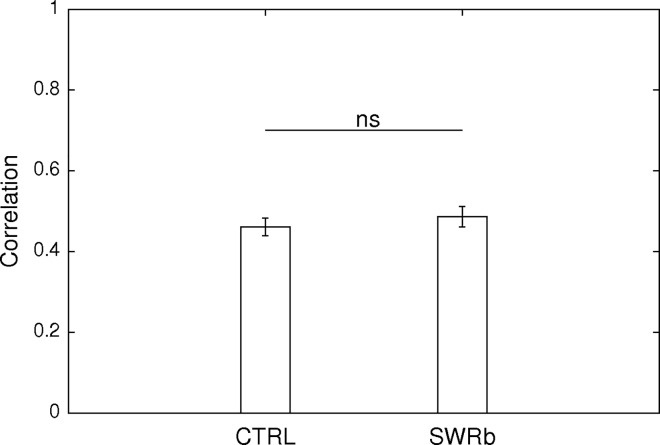
Stability at the population level. Cofiring similarity based on the joint firing of cell pairs between the first and the second exploration of the same environment on control days (1649 cell pairs, left bar) and SWR-blockade days (1176 cell pairs, right bar). The difference does not attain the threshold for significance (Z = -0.749; p = 0.454, Fisher’s Z-test).

Given that during SWR events firing patterns that have occurred during previous activity are replayed by CA1 cells, and SWR events are thought to reinforce the assembly association between the cells through such replay[18], it was reasonable to assume that this relationship between the CA1 cells will evolve differently upon SWR-blockade in spite of the stability and the minimal change observed at single cell level. Therefore, we have calculated the cofiring stability (Fig 11) by considering every possible CA1-CA1 pair in our dataset. To this end, exploration sessions were split up into temporal bins and cofiring vectors were computed for each cell pair. Subsequently, Pearson correlation of the cofiring vectors of the first and of the second exploration sessions were computed [19,20]. As shown in Fig 11, the pairwise cofiring similarity did not change significantly (Z = -0.749; p = 0.454, Fisher’s *Z*-test) between SWR-blockade and delayed control blockade, pointing to the conclusion that the disruption of the SWR events did not change the representation at the population level either.

## Discussion

In the present study we have optogenetically blocked SWR events with high temporal precision in sleep/rest and investigated the hippocampal (CA1) representation in the two flanking explorations of the same, initially novel environment. According to our results, neither parameters pertaining to single CA1 cells (sparsity, coherence, and Pearson-correlation of the firing rate maps) nor those rather pertaining to assemblies of CA1 cells (cofiring similarity) change upon disrupting SWR events in sleep/rest periods when compared to values measured in a control condition with non-coincident laser pulses. Previous reports have demonstrated that SWR events during sleeps intercalated between learning trials are needed for the improvement of behavioral performance in various tasks[9,10,12]. On the other hand, the ensemble activity of CA1 place cells in novel environments is thought to correlate with spatial memory and navigation[21]. Therefore, an explanation is needed as to why the CA1 code did not change after SWR blockade in our experiments.

It is possible that a larger cell population would be needed to demonstrate a weak but significant effect between the SWR-blockade and control conditions. We are confident that our optogenetic inhibition targeted a large enough region of the hippocampus because we have covered the dorsal CA1 with our layout of tetrodes and optic fibers (S4 Fig) and we have detected strong light-evoked local field potential responses on all tetrodes. It is possible, however, that with an increased yield of stable pyramidal units, yet hidden electrophysiological effects might be uncovered in the future even in simple open field experiments.

While not providing evidence for any role of sleep/rest SWR events in the stabilization of the code within the hippocampus, our study emphasizes the role of the contribution of sensory input for generating the hippocampal spatial code and the robustness of sensory-driven hippocampal cell firing. As reported recently, the so-called oriens-lacunosum moleculare interneurons can dynamically enforce a switch between two different inputs to the CA1, namely between that from CA3 and that from the entorhinal cortex [22]. It is reasonable to assume that inputs from the entorhinal cortex are more active althrough a task where no specific rules have to be retrieved, thus sensory input can be–as evidenced by our work–the main determining factor of the CA1 code at the second exploration as well.

How can one explain the disrupted spatial learning and recall as a result of SWR blockade if the same manipulation leaves spatial map representations intact? A candidate hypothesis would be that the main role of the SWR events is the transfer of the information to extrahippocampal areas and that this process is also underlying, to some extent, the behavioral impairment seen in different spatial tasks. Indeed, a very recent publication suggested a causal link between hippocampo-cortical coupling during SWR envelops in sleep/rest and improved performance in behavioral tasks [23]. Provided that this assumption is generally true, the blockade of SWR events could have an impact on memory and task-specific performance while it would not elicit any change at the level of the CA1 population code. At this point it has to be noted that in the hippocampus the offline increase in protein synthesis or offline transcriptional changes triggered by NMDA receptor activity during experience both have been shown to be required for the stabilization of the CA1 code[24–26] and this kind of off-line molecular changes constitute a prerequisite for learning[26,27]. However, none of these studies have linked such molecular events directly to the SWR envelope.

If the major role of the SWR events in slow-wave sleep were indeed the transfer of information to downstream brain areas, then explanation has to be provided as to how hippocampal assemblies representing specific aspects of the task are formed, and how intrahippocampal connections are reinforced. A reasonable assumption would posit that exploratory (waking) sharp wave ripple events (eSWR) [11] could play a major role in plastic processess leading to the reinforcement of new CA1 engrams.

As discussed in our introduction, evidence pointing to an alteration of SWR events in dementia is starting to accumulate[6–8]. Therefore, our optogenetic method to artificially disrupt SWR events could be used as a model to trigger cognitive deficits associated with dementia. Such a manipulation might open up new ways to test compounds intended to restore cognitive function. However, taken the results of our present work, CA1 population activity in simple open field paradigms and place field stability may not be a suitable biomarker in tests targeting restoration, given that SWR disruption in sleep/rest did not elicit a measurable change under these conditions.

## Materials and Methods

### Animals

Three C57/BL6 female mice expressing an Archaerhodopsin-EGFP in the pyramidal cells of the CA1 region were used in this study. Instead of viral expression we have used a permanently integrated transgene at a particular locus, to avoid the unstable kinetics of viral expression and the toxic overproduction of the engineered protein. To achieve the desired expression pattern we have crossed homozygous CaMKII-Cre animals from the Tg29 strain[14] with homozygous animals from the Ai35 strain[15] harboring a CAG-Flox-STOP-Flox-ss-Arch-EGFP. Both strains were purchased from the Jackson laboratory (JAX 005359 and 012735). In litters from such a cross, Cre recombinase is active in a subset of excitatory neurons, particularly those in the CA1 and dentate gyrus. We have checked the expression of the transgene in two unimplanted animals, and saw the expected regional distribution in the central nervous system. Photos of higher magnification confirmed that the exogenous fusion protein was restricted to the plasma membrane and was virtually absent from the cell bodies and nuclei. All procedures involving experimental animals were carried out in accordance with the *Austrian federal Law for experiments with animals* and under a project license approved by the Austrian Federal Ministry of Science, Research and Economics (project license number: BMWFW-66.018/0015-WF/V/3b/2014). After the experiments all mice were deeply anesthetized with pentobarbital and transcardially perfused with phosphate buffered saline followed by fixative (paraformaldehyde dissolved in PBS, 4% wt/vol).

### Optic ferrules and fibers

Poly-methyl-metacrilate (PMMA) optic fibers coupled to optic ferrules were ordered from Doric Lenses (www.doriclenses.com, catalogue number: MFC_240/250-0.63_15mm_MF1.25(GK)_C45), this type of fiber was flexible enough to be built into a microdrive, and half of its section protruding out from the microdrive was reinforced with polyimide tubing. The diameter of the fiber core was 240 μm, with cladding of 250 μm and its tip was conical (α = 45 °) which assured a smooth penetration in the brain tissue and together with the high numerical aperture (NA = 0.63) an efficient distribution of light over a large brain area. The custom-designed ferrule’s diameter was 1.25 mm, its total length 6.5 mm, and at a height of 1.6 mm it had 0.25 mm deep and 1.2 mm high diced grooves going around its perimeter. The 4 locking pieces (S1 Fig) could slide into these grooves to hold the ferrule firmly in the microdrive.

### Microdrive building

Microdrives were devised in collaboration with Axona (www.axona.com) and based on their already existing models. Briefly, a longer, bilateral microdrive model consisting of bottom, middle and top (connector) part with SOSSOS layout on both sides was developed where “S” stands for screw and “O” stands for optic ferrule (S1 and S3 Figs). Correspondingly, the optic ferrules were not positioned in the corner but closer to the center so that excessive bending of the optic fibers guided through the electrode holes could be avoided. Tetrodes were glued in steel cannulae held by movable shuttles into which screws made their thread at first assembly. Twin tetrodes of 12 μm were loaded in 7 shuttles, while a single tetrode of 17 μm was used in the remaining shuttle. All tetrodes were of tungsten wire covered by H-Formvar insulation covered with butyral bond coat (California Fine Wire, Grover Beach, CA). The connector part had a 5 × 13 layout, harboring 65 *Interconnets series 860* pins (www.mill-max.com) out of which 5 were used for the grounding at the occipital pole. The optic ferrules were immobilized in the drive (i) by a collar onto which the ferrules abutted and (ii) by a supplementary triangular piece that slided into a groove on the ferrule. Such triangular pieces were locked into their position by the insect pins which held the three parts of the drive together and served also as a track for the moving shuttles. Before loading the tetrodes, the optic ferrules and fibers were built into the drive and a 2 mm long polyimide-tube was pulled onto their protruding portion, leaving another ca. 2 mm of optic fiber uncovered at the bottom of the drive (S3 Fig). The tubing served to increase the stiffness of the optic fibers at implantation and therefore it was also glued to the bottom of the microdrive. Finally, tetrodes were lowered and cut, their tips were gold-plated to reduce electrode impedances to 250–300 kΩ and their tip was placed 150 μm above tip of the conical tip of the optic fibers.

### Surgery

Animals were anesthetized using 1% isofluorane in 1 dm^3^/min oxygen flow. Animals were initially anesthetized with 5% isoflurane, shaved, and then injected with 0.1 mg/kg buprenorphine and 10 mg/kg baytril. After shaving and disinfection, eyegel (GenTeal, Thea Pharma GmBH) was distributed over the eyeballs to avoid dehydration. An initial incision was made with a scalpel-blade, and the skin, together with the neck muscle, was held apart with surgical thread (VICRYL; Ethicon) instead of traditional clamps, and was regularly moistened with physiological solution. The skull bones were cleaned, the desired edges for bilateral craniotomy were marked by superficial drilling with drillbits of 0.5 mm (Hager and Meisinger GmBH, 1RF 005), and the following micro-screws (M1.0) were implanted after pre-drilling with drillbits of 0.7 mm (Hager and Meisinger GmBH, 1RF 007): 2 in each *os frontale*, 2 in each *os parietale* (laterally from the planned craniotomy), 2 in each *os occipitale*. The latter ones were targeted to touch the cerebellum and served as gound and reference electrodes. Subsequently a first layer of dental cement was used to establish contact between the skull bone and the screws, and then craniotomy was performed above the right (centered at AP = 2.08; ML = 1.65) and left (centered at AP = -2.08; ML = -1.65) dorsal portion of the hippocampus, the dura mater was removed and the electrodes were implanted together with the optic fibers by lowering the conical tip of the optic fibers down to a depth of exactly 1000 μm (upper half of the *stratum oriens*). The edge of the bottom part of the implanted drive was connected with the previously built cement wall using dental cement except on one side, after which the cannulae, the tubing of the optic fibers and the exposed neocortex were sealed with paraffin wax without letting the wax flow on the brain surface and finally the cement wall was closed. The wires from the occipital screws were soldered to the ground wires connected to the 5 ground pins of the microdrive. To close the wound, the frontal skin was stitched together using the surgical thread holding the skin apart, while the edge lateral and occipital skin was glued to the dental cement wall. During a one-week postoperative recovery period, Metacam (1.5 mg/ml meloxicam suspension, Boehringer-Ingelheim) was given in the drink and the skin around the implant was disinfected with Betain (10% povidone-iodine) and local anesthesia with Xylocain (2% lidocaine-hydrochloride, Astra-Zeneca) was performed if needed. During the second week after surgery the tetrodes were lowered into the CA1 regions of the hippocampus (to the stratum pyramidale) over a further period of around 4–6 days.

### Recording and analysis of the electrophysiological signal

Wide-band (0.4 Hz–9 kHz) recordings of local field potentials (LFPs) and multi-unit activity were amplified 1.000-fold via a 64-channel dacqUSB amplifier (Axona Ltd., St. Albans, Hertfordshire, UK) and continuously digitized at 24 kHz using a 64-channel analogue-to-digital converter built into the dacqUSB SYSTEM UNIT (Axona). To reduce cable movement artefacts, a 60-channel unity-gain preamplifier headstage was designed in house by wiring together 3 printed circuit boards that were driven by 5V and also had 5V sockets for two LED panels that were connected during the exploration sessions to enable 2 point tracking. Correspondingly, the position of the animal was continuously registered using an overhead camera and the tracking function of the dacqUSB SYSTEM UNIT in 2 point mode. Additionally, all exploration sessions were videotaped. The data were processed and analyzed off-line. Action potentials (APs) were first extracted from the raw recordings by computing the power in the 800–9000 Hz range in a sliding window (12.8 ms), then the APs with a power of > 5SD from the baseline mean were selected and their features were extracted using principal component analysis. The APs were subsequently segregated into clusters corresponding to putative single units using KlustaKwik 3.0 (http://klustakwik.sourceforge.net/). Finally such clusters were splitted, merged, and refined manually using Klusters (http://neurosuite.sourceforge.net/) to obtain units with clear refractory periods in their autocorrelograms. Only units independent from each other and showing stable activity throughout the 298 minutes experimental paradigm (Fig 1) were included. Pyramidal cells and interneurons in the CA1 region were discriminated by their autocorrelograms, overall firing rate and waveforms. Alltogether, 117 and 116 well separated CA1 pyramidal cells were included in the SWR-blockade condition and the control condition respectively. Analysis programs were written in C programming and Shell scripting languages and run under LINUX (CentOS 6) operating in an XWindow environment. Analyses were performed using custom made software. For statistics the STAT 5.4 UNIX software package 3 was used.

### Optogentic intervention

A COBOLT Jive 300 mW DPSS green (561 nm) laser (Omicron) was used as a light source together with a COBOLT controller device, and an AOM housing kit that enabled the reception of TTL signals from the Axona and the controlling voltage from an analogue controller device. The light was channeled out from the laser via a 1500 μm optic cable (Prizmatix, NA = 0.5) that was hung by the same counterbalanced pulley system as a the recording cable. A rotatory joint (Prizmatix) was wedged in between this cable and the 200 μm 4-branch fiber patch cord (Prizmatix) that had optic ferrules at the animal end. Typically at maximal laser intensity, 15 mW output was measured at each fiber end of the patch cord, and connecting an unimplanted microdrive to the cable via mating sleeves (Doric lenses) yielded ca. 10 mW of output at each conical fiber tip protruding out of the microdrive. For the 3 hour-long middle sleep this laser intensity was used, while for inihibition control sleep sessions the half-maximal laser pulses were applied (ca. 5 mW of output at each fiber tip in the *stratum oriens*) and the LFP responses decreased accordingly in this case.

### Behavior

Animals were kept on a standard (non-inverted) dark-light cycle, recordings started in the morning, and lasted 298 minutes long (Fig 1). Tetrode positions were fine-tuned on the previous evening using the hippocampal LFP as a guiding signal until large amplitude multi-unit activity was visible. First 20 minutes of presleep was recorded in the homecage, then the two LED panels enabling tracking were connected to the headstage, and the animal was exposed to a novel enclosure illuminated by dim lights for 24 minutes while positions and unit activity was recorded. In symmetric enclosures, a small symmetry breaking object was placed to avoid the mental rotation of the CA1 map at the second exposure. After the first exploration, the LED panels were uncoupled, and the animal was transferred back to the homecage with the recording cable still connected to the headstage and the optic cables connected via mating sleeves to the optic ferrules. During the subsequent three hours of sleep/rest periods containing predominantly slow-wave sleep epochs SWR events were blocked using the filtered (Bessel bandpass filter in the Axona) differential signal (on-line subtraction in the Axona) of a trigger channel and reference channel fed into the analogue ripple detector device (see below) that provided trigger for the laser. The first 20 minutes of each hour of this sleep session was recorded yielding 60 minutes of sleep data. On the control days, the filtered differential signal was similarly channeled into the ripple detector, the output of which was then fed back into a digital channel of the Axona dacqUSB SYSTEM UNIT, afterwards it was delayed using an Axona dacqBasic script (in total, 1.32 s of delay was introduced), and finally directed to a digital output channel of the Axona system to drive the laser. Recording days containing more than 5 minutes of continuous movement coupled with non-slow-wave-sleep electrophysiological signal in the middle 3h long sleep were excluded. Following the sleep session, the LED panels were plugged back into the headstage, the animals were re-exposed to the same enclosure in identical conditions for 24 minutes to record the second exploration. Finally the mouse was transferred back to the homecage, and 20 minutes of postsleep was recorded, followed by 30 minutes of laser control sleep with half-maximal laser power that was strictly non-saturating (continuous LFP signal could be recorded) and therefore the inhibition of individual cells could be established using this last session (Fig 3). Slight food restriction (half a pellet instead of food ad libitum, maximally down until 90% of the initial weight of the animal) was used during the night before the experimental day, to motivate the animal to explore the environment.

### Ripple detection

The signal from the filtered differential channel was fed into an analogue ripple detector device that contained a 4 pole filter using 150 Hz as the center frequency. The filtered signal was amplified with a gain of 10 and then compared to a trigger defined by trigger level potentiometer. For the wiring diagram of the device see Nokia et al. [28] When the signal was larger than the trigger level the ripple detector device emitted a 200 ms TTL pulse that was channeled either directly into the AOM housing kit controlling the laser (SWR-blockade condition) or into the Axona dacqUSB SYSTEM UNIT to introduce a delay before driving the laser (control condition).

## Supporting Information

S1 Fig Design of the microdrive. Bilateral optogenetic mouse microdrive is depicted with 4 screws for tetrodes and 2 optic fibers in each hemisphere. The optic cables are connected via the optic ferrules (only 2 are shown) locked into the microdrive.
(TIF)

S2 Fig Expression of the optogenetic actuator. Expression of the Arch-EGFP construct in the brain of mice obtained as a cross between Ai35 and Tg29 is shown in coronal sections. **(A)** whole hippocampus **(B)** CA1 region **(C)** CA1-CA2-C3 transition with strong expression in the stratum lucidum fibers **(D)** Strong expression in fibers terminating in the subiculum.
(TIF)

S3 Fig Microdrive building procedure. **(A)** optic fibers protruding from the bottom of an unloaded microdrive–note the yellow polyimide tubing **(B)** placing connector pins into a loaded microdrive **(C)** A fully finished microdrive viewed from the bottom–note that the cannulae are at the lowermost position therefore the optic fibers are not fully visible.
(TIF)

S4 Fig Arrangement of the optic fibers and tetrodes at the bottom part of the microdrive. Electrode holes were arranged in a regular hexagonal grid, with the shortest distance between the holes being 400 μm. Unused electrode holes are shown in white, those harboring an optic fiber in green, and those harboring a steel cannula for tetrodes in blue. The distance between contralateral optic fibers is also indicated along with the distance of the pairs from the bregma.
(TIF)
