# Correction: Optogenetically Blocking Sharp Wave Ripple Events in Sleep Does Not Interfere with the Formation of Stable Spatial Representation in the CA1 Area of the Hippocampus

**DOI:** 10.1371/journal.pone.0177565

**Published:** 2017-05-08

**Authors:** Krisztián A. Kovács, Joseph O’Neill, Philipp Schoenenberger, Markku Penttonen, Damaris K. Rangel Guerrero, Jozsef Csicsvari

The fifth author’s name is spelled incorrectly. The correct name is: Damaris K. Rangel Guerrero. The correct citation is: Kovács KA, O’Neill J, Schoenenberger P, Penttonen M, Rangel Guerrero DK, Csicsvari J (2016) Optogenetically Blocking Sharp Wave Ripple Events in Sleep Does Not Interfere with the Formation of Stable Spatial Representation in the CA1 Area of the Hippocampus. PLoS ONE 11(10): e0164675. https://doi.org/10.1371/journal.pone.0164675
