# Supplementary figures and images for: Optogenetically Blocking Sharp Wave Ripple Events in Sleep Does Not Interfere with the Formation of Stable Spatial Representation in the CA1 Area of the Hippocampus
Source: PLoS One. 2016 Oct 19;11(10):e0164675. doi: 10.1371/journal.pone.0164675 (PMC5070819; doi:10.1371/journal.pone.0164675)

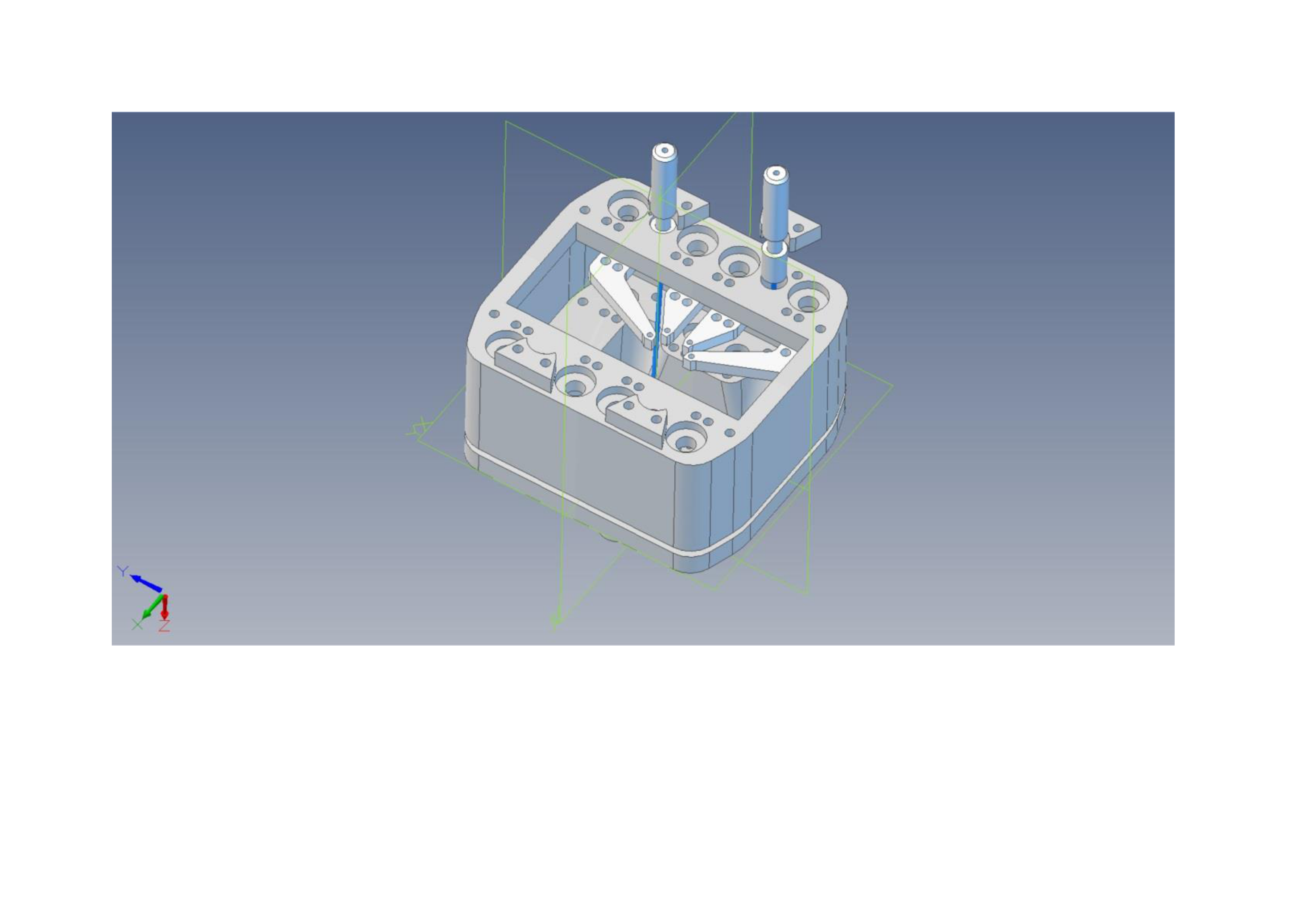

Supplement: S1 Fig — Bilateral optogenetic mouse microdrive is depicted with 4 screws for tetrodes and 2 optic fibers in each hemisphere. The optic cables are connected via the optic ferrules (only 2 are shown) locked into the microdrive. (TIF) [file pone.0164675.s001.tif]

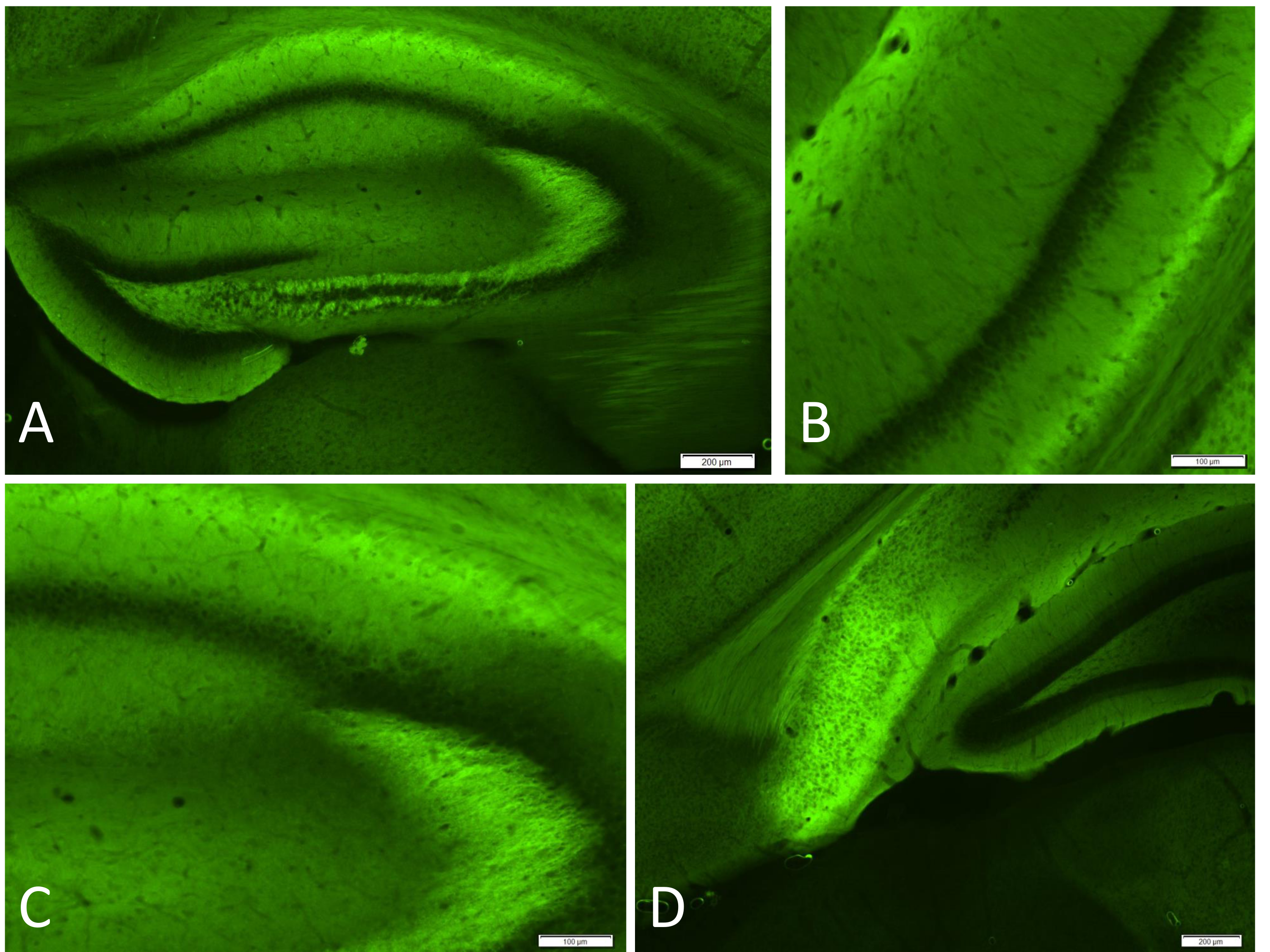

Supplement: S2 Fig — Expression of the Arch-EGFP construct in the brain of mice obtained as a cross between Ai35 and Tg29 is shown in coronal sections. (A) whole hippocampus (B) CA1 region (C) CA1-CA2-C3 transition with strong expression in the stratum lucidum fibers (D) Strong expression in fibers terminating in the subiculum. (TIF) [file pone.0164675.s002.tif]

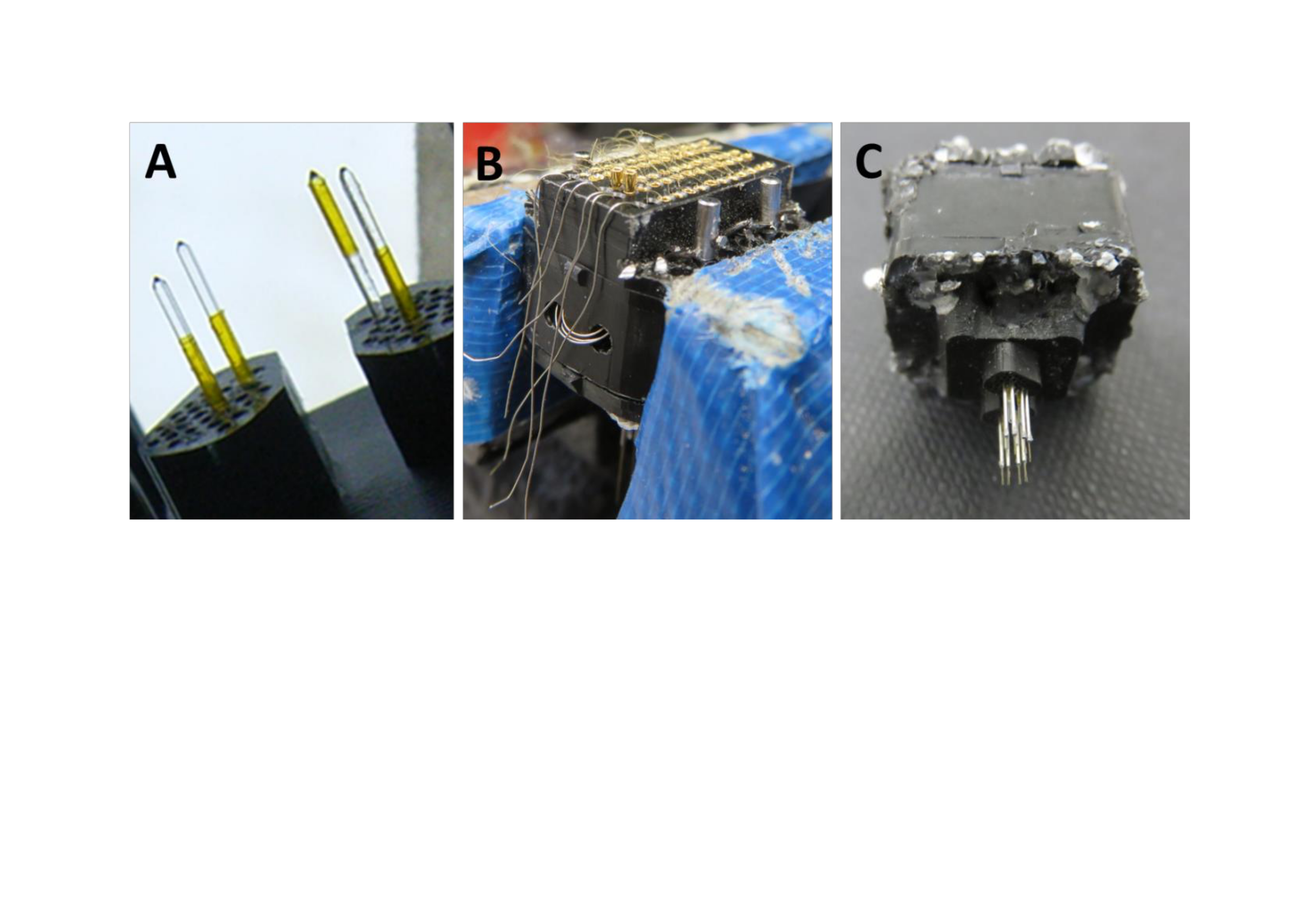

Supplement: S3 Fig — (A) optic fibers protruding from the bottom of an unloaded microdrive–note the yellow polyimide tubing (B) placing connector pins into a loaded microdrive (C) A fully finished microdrive viewed from the bottom–note that the cannulae are at the lowermost position therefore the optic fibers are not fully visible. (TIF) [file pone.0164675.s003.tif]

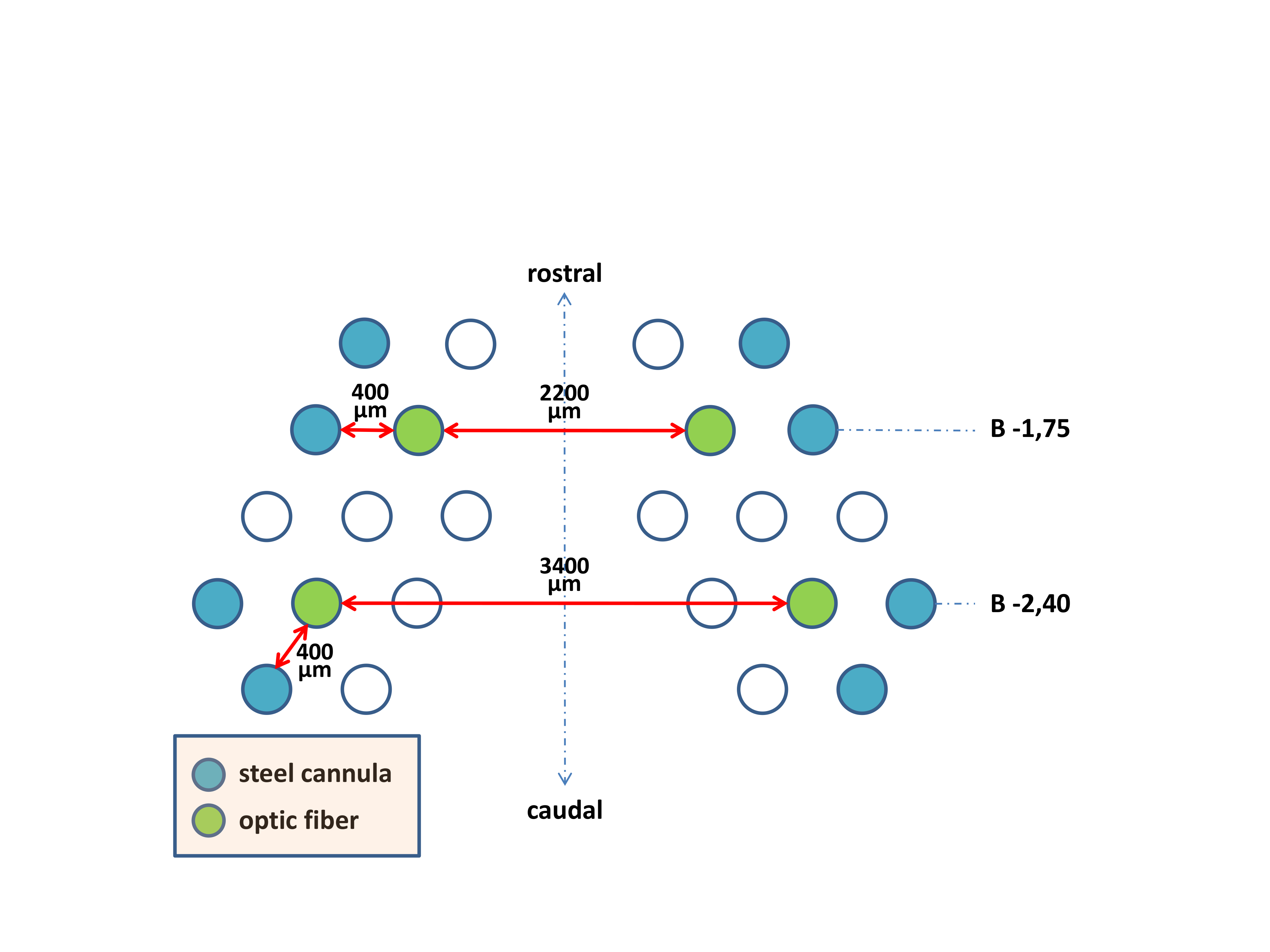

Supplement: S4 Fig — Electrode holes were arranged in a regular hexagonal grid, with the shortest distance between the holes being 400 μm. Unused electrode holes are shown in white, those harboring an optic fiber in green, and those harboring a steel cannula for tetrodes in blue. The distance between contralateral optic fibers is also indicated along with the distance of the pairs from the bregma. (TIF) [file pone.0164675.s004.tif]
